# Tau aggregation and its relation to selected forms of neuronal cell death

**DOI:** 10.1042/EBC20210030

**Published:** 2021-12-22

**Authors:** Aviva M. Tolkovsky, Maria Grazia Spillantini

**Affiliations:** Department of Clinical Neurosciences, Clifford Allbutt Building, University of Cambridge, Cambridge CB2 0AH, U.K.

**Keywords:** cell death, neurodegeneration, tau

## Abstract

How neurons die in neurodegenerative diseases is still unknown. The distinction between apoptosis as a genetically controlled mechanism, and necrosis, which was viewed as an unregulated process, has blurred with the ever-increasing number of necrotic-like death subroutines underpinned by genetically defined pathways. It is therefore pertinent to ask whether any of them apply to neuronal cell death in tauopathies. Although Alzheimer's disease (AD) is the most prevalent tauopathy, tauopathies comprise an array of over 30 diseases in which the cytoplasmic protein tau aggregates in neurons, and also, in some diseases, in glia. Animal models have sought to distil the contribution of tau aggregation to the cell death process but despite intensive research, no one mechanism of cell death has been unequivocally defined. The process of tau aggregation, and the fibrillar structures that form, touch on so many cellular functions that there is unlikely to be a simple linear pathway of death; as one is blocked another is likely to take the lead. It is timely to ask how far we have advanced into defining whether any of the molecular players in the new death subroutines participate in the death process. Here we briefly review the currently known cell death routines and explore what is known about their participation in tau aggregation-related cell death. We highlight the involvement of cell autonomous and the more recent non-cell autonomous pathways that may enhance tau-aggregate toxicity, and discuss recent findings that implicate microglial phagocytosis of live neurons with tau aggregates as a mechanism of death.

## Introduction

In 2022, it will be 50 years since the article by Kerr et al. [1] appeared that coined the phrase apoptosis. They described a mechanism of cell death that is characterised by cell condensation and fragmentation like the shedding of leaves or petals, and consequent phagocytic cell elimination, sometimes leaving an undigested residual body. The process was suggested to be ‘an active, inherently programmed phenomenon’ [2] as it was regulated by hormones and was observed to be prevalent in isolated cells during development. This was contrasted with necrosis, which was an indiscriminate process of cell lysis linked to inflammation. The remarkable conversion of apoptosis from an inherently morphological description into its detailed molecular underpinning has presented numerous potential therapeutic targets whose manipulation should allow either induction or inhibition of apoptosis and hence rescue of neuronal cell death in neurodegeneration. However, although occasional apoptotic footprints have been demonstrated in Alzheimer's disease (AD) and other tauopathies during neurodegeneration, or in animal models, it is clear that apoptosis is not the major mechanism of cell death in these diseases. On the other hand, interest in necrosis, previously considered an unregulated process, has blossomed and now encompasses several pathways underpinned by molecular mechanisms (all ending in ptosis although none seem to shed any parts of the cell) including: ferroptosis, pyropotsis, and necroptosis [3–5]. Additional necrotic types of death are mainly due to ATP depletion and include parthanatos, oncosis, autosis [4]. Intracellular digestion pathways have also been implicated, including lysosomal and autophagic cell death [3–5]. Finally, non-cell autonomous mechanisms include those instigated by phagocytosis of living neurons and possibly those instigated by immune cells that release granzyme B/perforin [6], the latter hardly explored in relation to neurodegenerative diseases. Another focus has been on degeneration of axons/synapses as a cause of retrograde degeneration. Table 1 summarises the features of the main cell death mechanisms sometimes also reported in tauopathies. Extensive discussions of the mechanisms of each pathway in relation to neuronal cell death and neurodegeneration can be found in recent reviews [4,5]. In this review, we reflect specifically on neuronal death in tauopathies.

**Table 1:** Summary of cell death mechanisms discussed in this review

Type of neuronal cell death	Initiators	Mediators	Executioners	Inhibitors	Outcome	References
Apoptosis	Death receptors, DNA damage, ROS, staurosporine	Intrinsic: pro-apoptotic Bcl2 members (Bax, Bim, Bak, Puma, Noxa), Bcl2 inhibitors (ABT737), extrinsic: upstream caspases 8/10	Apoptosome (cytochrome *c*/apaf1 activation (dATP- > dADP exchange) to caspase 9 and downstream caspases mitochondrial Smac/diablo inhibits IAP/xIAPs	Natural: anti-apoptotic Bcl2 members (Bcl2, BclxL, McL1) IAP/XIAP family of caspase inhibitors, caspase activity inhibitors (only temporary)	DNA breaks, nuclear condensation, loss of MOMP, exposure of PtdSer, removal by phagocytosis Eventual ATP depletion and secondary necrosis	[4,5,81]
Ferroptosis (oxytosis)	Fe^3+^ entry via transferrin receptor and conversion into Fe^2+^ lack of cysteine supply via Xc**^−^** transporter	Oxygen/hydroxyl radicals (via the Fenton reaction), and/or chemical GPx4 inhibitors (1S, 3E-RSL3)	Loss of glutathione, Lipid reactive oxygen species (LOOH, L–O], preferential oxidation of polyunsaturated fatty acids (PUFAs)	Glutathione peroxidase 4 (GPx4; up-regulation by Nrf2), antioxidants (ferrostatin-1, liproxstatin-1, vitamin E) iron chelators (Deferoxamine)	Plasma membrane lipid fragmentation, mitochondrial shrinkage/deformation of cristae, loss of ATP and NAD+, lysosomal membrane permeabilisation	[82–85]
Pyroptosis	Pattern recognition receptors (PRRs)/other signals (bacteria/toxins/dsDNA breaks)	Inflammasome (NLRP3 or its homologues/apoptosis-associated speck-like protein containing CARD (ASC/procaspase‐1) but also other mechanisms	Gasdermins (GSDMs), especially GSDMD, activated by caspase-1 cleavage downstream of inflammasome formation	Disulfiram (via Cys^191/192^ human/mouse in GSDMD)	Large pores with electrostatic filtering (preference for positively charged/neutral molecules) but no notable cell swelling can mediate IL-1b export	[86–90]
Necroptosis	Death receptors (TNF), caspase-8 inhibition	RIPK1/TRIF/ZBP1 binding to RIPK3 and phosphorylation of MLKL	Phospho-MLKL oligomerisation and translocation to plasma membrane	Necrostatins (RIPK1 inhibitors)	Pores in plasma membrane (and mitochondria/lysosomes); Na^+^ permeability, water influx, and osmotic swelling morphology	[36,37,91–93]
Parthanatos	Poly(ADP-ribose) polymerase 1 (PARP-1) hyperactivation	Apoptosis inducing factor (AIF)-dependent and microphage migration inhibitory factor (MIF)-dependent	DNA degradation	PARP inhibitors	Shrunken and condensed nuclei, membranes disintegrate, and cells become propidium iodide-positive within a few hours after the onset	[94,95]
Autosis^*^	Hyper autophagy activation	Ions	Osmotic imbalance	Autosis - NaKATpase inhibitors, e.g. ouabain or autophagy inhibitors	Nuclear shrinkage; focal separation of inner and outer nuclear membranes with focal expansion of perinuclear space Extensive cytoplasmic vacuolisation increased adhesion	[96,97]
Primary phagocytosis^†^	Inflammation, stress LPS activation of microglia; ROS induced by tau aggregates	Phosphatidylserine/calreticulin exposure on the target cell	Opsonins (MFGE8, Gas6, protein S) made by phagocyte	CD47, excess AnnexinV or synaptotagmin C2 domain	Phagocytosis of live cell, inhibition of phagocytosis leaves behind a live cell	[50,57,98,99]
Perforin/Granzyme B	Cytotoxic T cells (CTLs) and natural killer (NK) cells	Ionic pores	Granzyme proteolysis and other activities	unknown	Stored as granules. Perforin creates ionic pores in the target cell, granzymes facilitate cell death by various mechanisms	[6,100,101]

* Forms of lysosome-dependent cell death, and autophagy-dependent cell death are not included because their molecular mechanisms are not proven except in invertebrates.

† Also named ‘phagoptosis’ although there is no ‘ptosis’ element in this form of death.

## Tauopathies

Tau (an acronym of ‘tubule-associated unit’) in adult human brain comprises six isoforms that result from alternative splicing of the *MAPT* gene; splicing of exons 2 and 3 lead to the absence or presence of one or two N-terminal repeats (labelled 0N, 1N, or 2N), and splicing of exon 10 results in tau containing 3-repeat or 4-repeat units (3R, 4R) that are responsible for binding and stabilising microtubules besides other functions [7]. In disease, tau undergoes numerous post-translational modifications beginning with hyperphosphorylations that repel tau from the microtubules and contribute to its misfolding, eventually leading to tau aggregation and fibrillisation [8]. A recent study of post-translational modifications of tau from AD patients using mass spectrometry has proposed that following hyperphosphorylation, tau undergoes acetylations, methylations, and ubquitinations that facilitate the formation of fibrils [9]. This study also showed a great diversity in the spectrum of post-translational modifications in different patients, although all were diagnosed with AD.

Aggregation of the protein tau from a soluble unfolded state to an insoluble, β-sheet-rich filamentous structure underlies numerous human neurodegenerative diseases known as tauopathies [10,11]. These include AD, frontotemporal dementias, Pick's disease, progressive supranuclear palsy (PSP), corticobasal degeneration, chronic traumatic encephalopathy, and argyrophilic brain disease. The presence of intraneuronal aggregates of tau best correlate with the neuronal cell death that is associated with the clinical signs and symptoms of diseases such as AD, PSP, and Pick's disease [12]. The mechanism of cell death induced by tau is not well understood. Tau misfolds into distinct fibrillar forms depending on the specific tauopathy [13]. Different filamentous forms of tau can template specific pathological conformations on to naïve monomers through a mechanism of prion-like spreading [14]. Analysis of disease progression suggests that misfolded tau is released as seeds through synaptic connections, because pathology proceeds progressively via anatomically connected regions [15,16]. Since cryo-EM studies have shown that tau fibril structures differ between each type of tauopathy [17], it may not be surprising if the mechanisms of cell death will also be diverse. The exact structure of tau as well as the millieu of the cell may dictate which death pathway is followed. Tau toxicity studies *in vitro* have mainly relied on tau fibrils formed by co-incubation with heparin but given the recent report that heparin-aggregated tau does not resemble fibrils extracted from tauopathies [18], it is difficult to conclude that similar mechanisms occur in human disease. Indeed, non-neuronal cells containing tau aggregates formed by seeding can be amplified into clones, demonstrating that such tau aggregates in these models are not inherently toxic [19].

It has been hypothesised that oligomeric tau is the toxic species whereas filamentous tau is benign or is even formed in an attempt to sequester tau away from toxic substrates. A recent study of transgenic mice overexpressing the human mutant P301S 0N4R isoform of tau in neurons [20], where neuronal cell death in the cortex and motor neurons in the brain and spinal cord is prevalent, suggests that filamentous aggregated tau is the toxic species [21], although this does not preclude the co-presence of oligomers in the same neurons. Antibodies that detect oligomers [22] in our hands failed to find such oligomers in neurons expressing P301S tau unless they also contained insoluble tau aggregates (unpublished data). Indeed it has been hypothesised from a kinetic analysis that the formation of sarkosyl-insoluble tau species must also include a fragmentation process [23], which could give rise to secondary oligomers. A recent study using a tau-proximity ligation assay has suggested that tau–tau interactions occur before detection of formation of tau aggregates in AD (at Braak stage I/II ), with positive structures occuring in both the neuropile and in neuronal cell bodies [24] but the sizes of the multimers and their purity remains to be determined.

## Tau aggregation or pathology and apoptosis

Apoptosis was studied extensively as a possible mode of death in AD, but less so in pure tauopathies. Most studies of apoptosis were directed at detecting elevated levels of executioner caspases (mainly caspase 3) but caspases have numerous non-apoptotic roles in the nervous system [25]. Of interest is the observation that caspases play a key role in synapse pruning by microglia, by promoting the exposure of phosphatidylserine (PtdSer) and recruitment of C1q [26–28]. Apoptosis is a rapid cell degradation process with clear molecular and morphological correlates, but because apoptotic cells are rapidly cleared, it is difficult to confirm apoptotic death in neurons containing tau aggregates/filaments and in diseases that last for decades. Nevertheless, it has been suggested that executioner caspases play a role in generating more aggregation-prone tau after tau cleavage at Aspartyl 421 (D421) residue. However, in a study on rTg4510 tau transgenic mice, caspase activation preceded tau tangle formation by hours to days yet neurons remained alive after a new tangle formed within the same neurons [29], thus dissociating caspase activity from apoptosis, which is normally executed within 24–48 h of caspase activation. Moreover, in the P301S tau mouse model, hardly any active caspase 3 or its substrate, cleaved fodrin (an enodgenous intracellular reporter used to demonstrate productive caspase activity), was detected in regions where neuronal death exceeds 50% [20]. Truncated D421 tau was also very low in dispersed filaments from AD and FTDP-17 brains and appeared late in the progression of the disease. Although the possibility that there are undetectable small amounts of D421-truncated tau cannot be excluded, these data suggest that truncation at D421 is not necessary for the assembly of tau into filaments [30]. Interestingly, phosphorylation of tau at Ser422 (a site modified specifically in tauopathies [9]), appears to prevent caspase cleavage [31], indicating that caspase activation deduced from staining by antibodies against the active form does not necessarily result in tau cleavage to a more aggregation-prone form of tau or cell death.

## Tau aggregation or pathology and regulated types of necrosis – focus on necroptosis

While there is some indirect evidence implicating tau and activation of regulated forms of necrosis like pyroptosis [32], parthanatos [33] and ferroptosis [34,35], reviewed in [5], necroptosis has been more directly implicated in AD. Initial studies were based on the elevated expression of RIPK1 that correlated with Braak staging [36]. RIPK1 is activated by TNF-α and hence is also implicated in inflammatory processes in AD, this so far has been mainly associated with β-amyloid deposition and disposal [37]. Importantly, the execution of necroptosis is via pores formed by P-MLKL so it is necessary to demonstrate that P-MLML has ben recruited to the plasma membrane and not just demonstrate expression of the upstream kinases.

Recently, it was reported that necrosome components accumulate alongside tau inside granulovacuolar bodies (GvBs) in AD [38,39], correlating with tau pathology and neuronal loss, but not with amyloid plaques. The RIPK1 inhibitor, Nec-1, was also reported to reduce tau phosphorylation at Ser^199^ alongside cognitive functions in APP/PS1 mice that produce β-amyloid [40]. GvBs are associated with many classes of degenerating neurons that contain tau neurofibrillary tangles in AD and other tauopthies [41], hence the hypothesis that tau-associated cell death could occurs through granuolvacuolar degeneration (GvD) [42]. Phospho-tau has been reported to be contained within GvB, alongside an unusually large cohort of other phosphoproteins and kinases such as CK1∂ [43]. GvB vacuoles were suggested to be late-stage autophagic organelles [44] and recently GvBs have been identified as specific neuronal lysosomes that form as a direct result of tau aggregation (induced by seeding) due to impaired vesicle trafficking [45]. Since tau fibril formation is dependent on tau concentration, sequestering tau in vesicles might contribute to increased tau toxicity or reduce it, as hypothesised in [46]. Possible implication of lysosomal and autophagic mechanisms of cell death has been an area of debate, especially as these could theoretically lead to clearance of pathological forms of tau [47,48]. One problem is how to interpret a change in expression of markers that participate in these processes such as the autophagosomal marker LC3II, whose accumulation could either be due to *bona fide* activation of autophagic flux, or to a block in lysosomal proteolytic function, resulting in autophagosome accumulation but unproductive autophagy [49].

Studying the mechanism of death of dorsal root ganglion (DRG) neurons containing insoluble tau aggregates cultured from adult P301S tau mice, we found that neuronal loss was not ameliorated by incubation with Nec-1 nor did it differ from the rate of loss in the presence of the inactive isomer Nec-1i [50]. The implication of coexistence of tau aggregates and necrosome in relation to pathology and neuron death remain to be deciphered.

## Tau aggregation or pathology and non-cell autonomous death mechanisms

Although the participation of necroptosis in tau-related neuronal cell death is still debated, an interesting avenue is the finding that necroptosis is a major mechanism that is involved in microglial-mediated cell death. Microglia and astrocytes have now been implicated in mediating non-cell autonomous neuronal cell death in several neurodegenerative disease models [51–53]. *In vitro*, inhibition of caspase 8 (which complexes with RIPK1) – but not caspase 3 – was sufficient to rescue live neurons that would otherwise have been killed by microglial phagocytosis, thus revealing a new mechanism of neuronal cell death called primary phagocytosis. In this example, primary phagocytosis was due to an interplay of oxidative stimuli that activated the microglia and caused neurons to expose the ‘eat-me’ signal PtdSer [54,55].

It has long been known that apoptotic cells display PtdSer and are thereby eliminated by phagocytosis without producing inflammation, unlike forms of unregulated necrosis. The cardinal sign of primary phagocytosis is that inhibition of microglia in this context rescues live neurons, whereas dead neurons accumulate in the case of apoptotic phagocytosis. Such mechanisms also contribute to synapse elimination in health and disease [26]. We have recently reported that neurons with insoluble tau aggregates that are cultured from P301S tau mice are eliminated by primary phagocytosis by microglia, and not by apoptosis or necroptosis. Tau aggregates caused the neurons to display PtdSer because of reactive oxygen species (ROS) induction [50], and contact with microglia caused them to release opsonins that enabled the phagocytic process. Interestingly, these microglia released tau aggregates they had ingested with the neurons, while releasing other senescence-associated paracrine factors that caused other microglia to become hypophagocytic, which could exacerbate the development of tauopathy, tau toxicity, and cell death [56,57]. Interestingly, microglial senescence has been associated with tau pathology and neurodegeneration in AD and other tauopathies [58,59] and tau aggregate-associated neuronal senescence has also been implicated in neurodegeneration [60], although the mechanism of neuronal death has not been defined. While aggregated tau alongside neuronal nuclei were found in the microglia in the brains of these mice, it is possible that not all the neurons with aggregated tau die by this process. Indeed, some motor neurons that contain tau aggregates in the brain and spinal cord of P301S tau mice and in tauopathies show signs of nuclear condensation and cytoplasmic swelling, hallmarks of late necrosis, but whether this is a stage *en route* to phagocytosis remains to be resolved. Studies in PS19 tau mice corroborate the active role of microglia in neurodegeneration and in synapse removal but how the neurons die in these models remains to be described [61–64].

Astrocytes have also been suggested to promote neuronal death, for example [65,66]). In many pure tauopathies, tau aggregates appear in astrocytes, leading to the hypothesis that astrocytes are sources of tau spreading and loss of neuronal support, thus contributing to tau pathology [67]. In ALS, astrocytes were suggested to kill neurons by necroptosis although recent work suggests that other mechanisms prevail (for example [68]). In the P301S tau mice, we found that neuron death is reduced by transplantation of neural precursor-derived astrocytes from wildtype (wt) mice [69]. Moreover, astrocytes cultured from P301S or P301L tau mice were deficient in providing neuroprotection and synaptogenesis compared with wt astrocytes [70], and supplementation of P301S astrocyte-derived conditioned medium was sufficient to rescue both neuron survival and synaptogenesis. That P301S tau neurons can cause astrocytes to lose their neuroprotective functions was suggested by the loss of several important functional markers induced in astrocytes by their interaction with healthy wt neurons [71]. Considering possible therapeutic interventions, it is important to consider not only how neurons die but also what factors might be used to boost their survival. The question has arisen regarding why only some neurons are vulnerable to tau-related diseases, and why these populations differ in different tauopathies. Propagation of tau between connected neurons might explain part of this selectivity but recent reports suggest that vulnerable neurons that die are more sensitive to development of tau pathology, possibly linked to loss of neuroprotective functions by astrocytes [72]. In P301S tau mice, injection of α-B crystallin (HspB5), a member of a small chaperone family of proteins, reduced neuronal cell death while boosting production of neurotrophic factors from astrocytes in cooperation with factors released by stimulation of microglia with HspB5 [73].

With the increase in identifying inflammatory mechanisms in long-term diseases like AD and other tauopathies, it may be pertinent to ask whether cytotoxic T cells are involved in some cell death events in tauopathies. Cytotoxic T cells are cells that recognise antigens presented usually due to viral infections, and were recently reported to invade the CNS during inflammation caused by COVID-19 [74]. Some vulnerable neurons were also reported to increase antigen-presenting MHC-I on their surface and this was related to exacerbation of tau pathology [75]. While perforin/granzyme B in tauopathies has not been reported, we thought it is important to include this mechanism of cell death in Table 1, especially if secondary infections occur that escape treatment.

## Are the mechanisms uncovered in mouse models matched by those in human disease?

**Figure 1:**
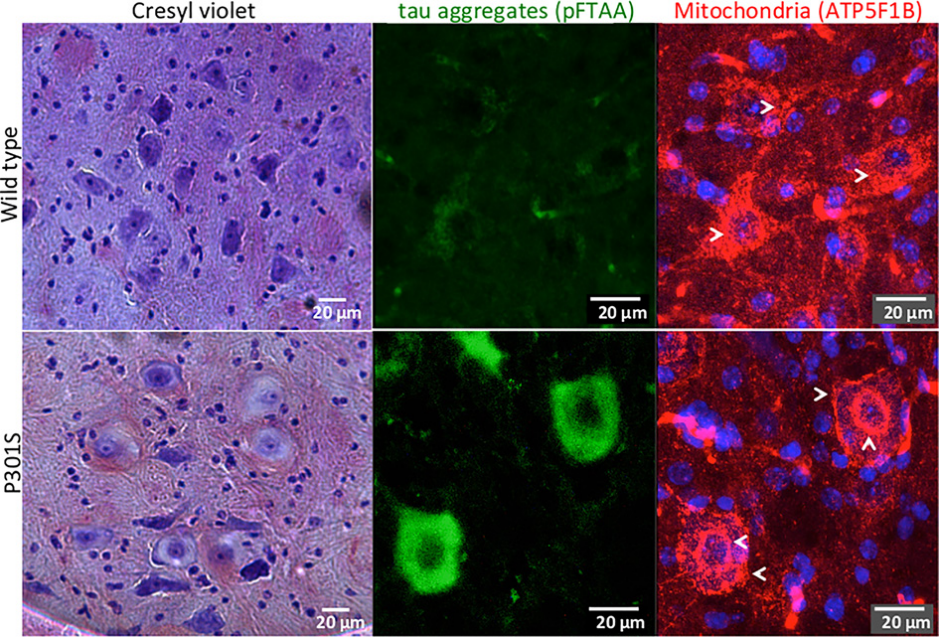
Neurons from P301S tau mice containing aggregated tau showing possible morphological features of ballooned neurons Brain stem sections from the facial nucleus region of 5-month-old wt C57 or P301S tau mice. Left column, stained with Cresyl Violet (note swollen and acentric nuclei in the sample from P301S tau mice). Middle and right columns, confocal Z stacks of the same region stained with pFTAA (green), a dye that detects tau aggregates with high affinity [102] and an antibody against the mitochondrial inner membrane protein ATP synthase β (ATP5F1B, red). Nuclei are in blue. Note the loss and rearrangement of remaining mitochondria around the nuclear and plasma membranes in neurons with tau aggregates (indicated by arrows).

When symptoms appear in tauopathies the neurodegenerative process has possibly gone on for decades and it is very difficult in postmortem human brain to clearly identify how the neurons have died. Both mouse models and cellular models have been developed reproducing tau aggregation to identify possible mechanisms of neuronal death related to tau aggregation. However transgenic models for example offer a speeded up process in that a mouse lives only for a few years so it is important, when possible, to validate the finding in mouse in the human brain before any conclusions can be reached. Neuropathological features of postmortem human brain can also provide suggestions for mechanisms of neuronal death, for example, ‘ballooned neurons’, observed in corticobasal degeneration, PSP, Pick's disease, and argyrophilic brain diseases [76], have been associated with neuronal death due to axon degeneration [77]. This still enigmatic form of death is characterised by enlargement of the neuronal cell body with cytoplasmic dilation, acentric nuclei and accumulation of neurofilament and staining for aβ-crystallin; in some cases, neurons also contain tau aggregates [78,79]. In the P301S tau mice, similar neurons are found in areas with neuronal death such as the spinal cord, where either swollen or pyknotic neurons were reported [20]. In these P301S tau mice, we correlated the presence of aggregated tau with the morphological features of ballooned neurons and found that the swollen cytoplasm and nuclei were poorly stained with the nucleic acid dye Cresyl Violet. A rearrangement of mitochondria around the cell periphery and the nuclear membrane was also present (unpublished, Figure 1). It will be interesting to determine how the redistribution of mitochondria and lack of nucleic acid staining is related to the neuronal death process.

**Figure 2:**
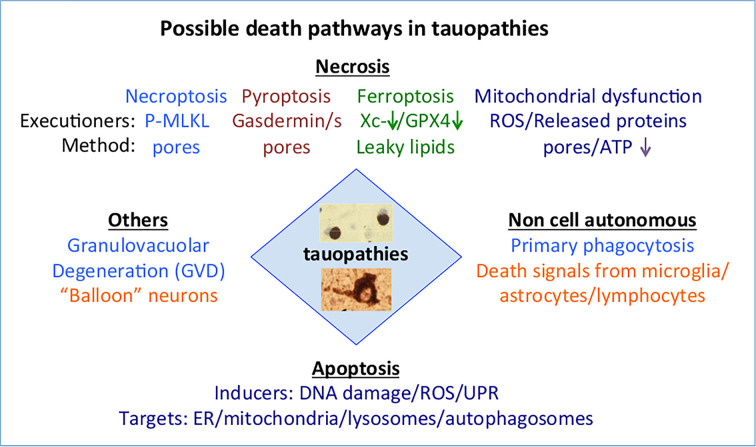
Scheme outlining the possible relationship between tauopathies and the various forms of death described in this review Different forms of tau aggregates/fibrils are found in different tauopathies but whether these are associated with specific neuronal cell death mechanisms remains to be determined. Two forms are illustrated in the central diamond-labelled tauopathies, the upper image shows two Pick bodies, while the lower image shows a neuronal tau tangle from an AD brain. Staining is with the anti-phospho-Ser^202/205^ antibody AT8 followed by immunohistochemistry with diaminobenzidine (DAB). The different colours under the various headings indicate separate mechanisms. Abbreviations: GPX4, glutathione peroxidase 4; ER, endoplasmic reticulum; UPR, unfolded protein response (induced by ER stress); Xc^−^, the glutamate/cystine transporter encoded by the *SLC7A11* gene.

Remaining questions abound. Is it naïve to expect that the mechanisms of neuronal cell death in tauopathies follow an exclusive pathway? Different mechanisms of cell death overlap in many of their biochemical pathways (in particular those affecting the integrity of the membranes in mitochondria, lysosomes, the endoplasmic reticulum (ER), and the plasma membranes). Organelle dysfunction can be triggered sequentially by one system going awry causing a step that is not part of a specific subroutine to be mobilised. In fixed postmortem tissue, we cannot measure flux events like calcium, which can activate proteases and cause intracellular dyshomoeostasis of all the internal organelles (mitochondria, ER, lysosomes, and nuclei), causing them not only to lose function but also to release further toxic factors. A second question is whether it is possible to define the cell death routines that indicate possible points of interventions. In this process, it may be useful to consider the concept of death commitment point, the point beyond which an intervention will not improve the prospect of survival. In apoptosis, it is clear that blocking caspases downstream of mitochondrial permeability transition is unlikely to rescue the cells unless mitochondria can be repaired, for example, by refilling them with cytochrome *c* [80]. However, preventing a dysfunctional neuron from dying without addressing the toxic cause may not rescue the dysfunction in the disease.

## Summary

A scheme showing how tauopathies relate to the various forms of death under discussion is shown in Figure 2.

- Neuronal cell death in tauopathies is not a simple linear process via one exclusive mechanism.
- Some forms of neuronal death involve other types of cells besides neurons.
- Identifying toxic species of tau will help unravel the downstream events that lead to death.
- Rescue of neurons requires identifying the most upstream step that defines death commitment point; this step can then be targeted for treatment.

## Open Access

Open access for this article was enabled by the participation of University of Cambridge in an all-inclusive *Read & Publish* pilot with Portland Press and the Biochemical Society under a transformative agreement with JISC.

## Abbreviations

AD: Alzheimer's disease
ALS: Amyotrophic Lateral Sclerosis
CNS: central nervous system
Cryo-EM: cryogenic electron microscopy
ER: endoplasmic reticulum
GvB: granulovacuolar body
PSP: progressive supranuclear palsy
PtdSer: phosphatidylserine
tau: tubule-associated unit
wt: wildtype
